# Influence of host iron status on *Plasmodium falciparum* infection

**DOI:** 10.3389/fphar.2014.00084

**Published:** 2014-05-06

**Authors:** Martha A. Clark, Morgan M. Goheen, Carla Cerami

**Affiliations:** ^1^Microbiology and Immunology, University of North CarolinaChapel Hill, NC, USA; ^2^Epidemiology, Gillings School of Global Public Health, University of North Carolina at Chapel HillChapel Hill, NC, USA

**Keywords:** malaria, iron, iron deficiency anemia, *Plasmodium falciparum*, iron supplementation

## Abstract

Iron deficiency affects one quarter of the world's population and causes significant morbidity, including detrimental effects on immune function and cognitive development. Accordingly, the World Health Organization (WHO) recommends routine iron supplementation in children and adults in areas with a high prevalence of iron deficiency. However, a large body of clinical and epidemiological evidence has accumulated which clearly demonstrates that host iron deficiency is protective against falciparum malaria and that host iron supplementation may increase the risk of malaria. Although many effective antimalarial treatments and preventive measures are available, malaria remains a significant public health problem, in part because the mechanisms of malaria pathogenesis remain obscured by the complexity of the relationships that exist between parasite virulence factors, host susceptibility traits, and the immune responses that modulate disease. Here we review (i) the clinical and epidemiological data that describes the relationship between host iron status and malaria infection and (ii) the current understanding of the biological basis for these clinical and epidemiological observations.

Iron deficiency and malaria are significant co-morbidities in large portions of the developing world, and both maladies disproportionately affect pregnant women and children. Malaria causes an estimated 250 million infections and 500,000 deaths annually. Iron deficiency is estimated to affect one quarter of the world's populations causing substantial morbidity. Fortunately, iron deficiency is easily treated with iron supplementation (Okebe et al., 2011). Accordingly the World Health Organization (WHO) recommends routine iron supplementation for children and adults in areas with high prevalence of iron deficiency (Haider et al., 2013; Low et al., 2013). However, the wisdom of universal iron supplementation campaigns in malaria endemic regions has recently been questioned due to clinical evidence that suggests iron deficiency protects against malaria, and that iron supplementation of women and children may increase the incidence of malaria when given without malaria prophylaxis or access to adequate health care (Nyakeriga et al., 2004; Sazawal et al., 2006; Tielsch et al., 2006; Kabyemela et al., 2008; Senga et al., 2011; Veenemans et al., 2011; Gwamaka et al., 2012; Jonker et al., 2012; Esan et al., 2013; Zlotkin et al., 2013). This situation has created a dilemma for health policy makers and health care workers in malaria endemic regions of the world (Prentice et al., 2013).

Despite these clinical and epidemiological studies, the extent to which the human host's iron status affects risk to and severity of malaria infection is unknown. Differences in study design and confounding factors (such as acquired immunity to malaria and hemoglobinopathies) have made the clinical and epidemiological studies difficult to interpret (Prentice et al., 2007). Furthermore, though iron and malaria have been and continue to be studied the exact biological relationship between host iron and malaria virulence remains largely unclear.

## Iron deficiency and iron deficiency anemia

Iron deficiency is a condition in which there is insufficient iron in the body to maintain normal physiologic functions. Iron deficiency can be categorized into three stages: iron deficiency without anemia, iron deficiency with mild anemia, and iron deficiency with severe anemia. Iron deficiency anemia occurs when iron stores are exhausted and the supply of iron to tissue is compromised; this condition is defined as anemia with biochemical evidence of iron deficiency. Iron deficiency is most prevalent and severe in young children and women of reproductive age, but can also occur in older children, adolescents, adult men, and the elderly. It is estimated that 50% of pregnant women and 40% of preschool children in the developing world are iron deficient (WHO | Assessing the iron status of populations, 2007; Kassebaum et al., 2014). Often, iron deficiency develops slowly and is not clinically diagnosed until severe anemia is apparent (Stoltzfus, 2003).

Studies suggest that iron deficiency impairs the growth, cognition, and neurological development of children from infancy through adolescence, impairs immune function, and is associated with increased morbidity rates (De-Regil et al., 2011, 2013; Wang et al., 2013). Iron deficiency during pregnancy is associated with multiple adverse outcomes for both mother and infant, including increased risk of hemorrhage, sepsis, maternal mortality, perinatal mortality, and low birth weight (Peña-Rosas et al., 2012a,b). Iron deficiency anemia can be a direct cause of death or contribute indirectly. For example, during child birth an anemic mother cannot afford to lose more than 150 mL of blood, compared with a healthy mother who can lose up to 1 liter of blood and still survive. Thus, the WHO recommends iron supplementation for all men, women, and children in areas where malnutrition is prevalent (WHO | Guidelines on food fortification with micronutrients, 2006).

Host iron metabolism is intimately linked to the host response to infection and inflammation. In the face of infection and inflammation, the human host protein hepcidin becomes elevated and initiates signaling which results in reduced iron absorption into the body along with the redistribution of body iron stores. As a consequence many of the biomarkers utilized to assess host iron status are sensitive to both iron as well as infection. For example, low serum ferritin (serum ferritin reflects total body iron reservoirs) is indicative of iron deficiency. However, ferritin is also an acute phase protein which is elevated in the context of infection, and as a result is not a reliable marker of human iron status in the presence of infection or inflammation. Like serum ferritin, transferrin saturation and transferrin receptor levels are biochemical markers of human iron status that are also sensitive to infection and inflammation. As a result evaluating an individual's iron status during an infection has proven difficult (Aguilar et al., 2012), and the scientific community has struggled to establish formal guidelines.

## Malaria

In 2012 malaria caused an estimated 207 million infections and over 600,000 deaths; 90% of these deaths occurred in sub-Saharan Africa, and 77% occurred in children under five (WHO | World Malaria Report, 2013). At least five species of the eukaryotic Apicomplexan parasite from the genus *Plasmodium* cause malaria in humans with *Plasmodium falciparum* being the most common and deadly. Following the bite of a malaria parasite infected mosquito, the sporozoite stage of the parasite enters the bloodstream and travels to the liver, where it subsequently infects liver hepatocytes. Malaria replication in the liver is asymptomatic. Next, the merozoite form of the parasite leaves the liver and enters into circulation to infect host red blood cells (RBCs). During the erythrocytic stage of infection, the parasite repeatedly invades, replicates within, and egresses from host RBCs. This erythrocytic stage of infection is responsible for all symptoms of disease (Miller et al., 2013), and the severity of disease is directly associated with parasite burden (Chotivanich et al., 2000; Dondorp et al., 2005).

A wide range of symptoms can be observed in malaria patients. Clinically however, malaria is categorized as either uncomplicated or complicated. Complicated malaria is further divided into three overlapping syndromes: cerebral malaria, severe anemia, and metabolic acidosis. The clinical syndrome observed in each individual patient is influenced by multiple variables: parasite species, host immune status, and genetic background, as well as the use and timing of antimalarial drugs (Taylor et al., 2010).

## Clinical studies linking iron and malaria infection

Host iron has received significant attention at the clinical level as a major factor that may regulate malaria virulence. The results of clinical studies conducted prior to 2002 which examined the relationship between host iron status and malaria risk are reviewed in three meta-analyses (Shankar, 2000; Oppenheimer, 2001; Gera and Sachdev, 2002). In the interim, two large iron supplementation trials as well as several smaller clinical studies have shed further light on the relationship between host iron status and malaria infection (Table 1). Clinical trials that have examined the relationship between host iron and malaria fall into two basic categories: those that observe the rate of malaria in individuals with iron deficiency anemia, and those that look at the rate of malaria infection in individuals given iron supplementation. Differences in study design exist within both study types, and include: the definition of study participant iron status, the administration of iron alone or with folate, and access to health care. Despite these differences, assessment of the outcome of the clinical studies has led to the general consensus that iron deficiency is protective against malaria, and iron supplementation increases malaria risk in the absence of access to adequate health care (Prentice and Cox, 2012; Spottiswoode et al., 2012; Stoltzfus, 2012).

**Table 1:** **Summary of clinical studies on iron deficiency, iron supplementation, and malaria**.

**References**	**Study design**	**Population**	**Country, malaria info**	**Results**
**CHILDREN—INTERVENTIONAL STUDIES**				
Sazawal et al., 2006	Randomized placebo controlled	7950 children given iron and folic acid	Zanzibar, intense malaria transmission	Trial stopped early because of safety concerns. Those who received iron and folic acid with or without zinc were 12% (95% CI 2–23, *p* = 0.02) more likely to die or need hospital treatment for an adverse event and 11% (95% CI 1–23, *p* = 0.03) more likely to be admitted to hospital; there were also 15% (95% CI 7–41, *p* = 0.19) more deaths in these groups
		8120 children given iron, folic acid and zinc		
		8006 control children		
		Ages 1–35 months		
Tielsch et al., 2006	Randomized placebo controlled	8337 children given iron and folic acid	Nepal, no malaria	Daily supplementation of young children in southern Nepal with iron and folic acid with or without zinc had no effect on their risk of death, but might protect against diarrhea, dysentery, and acute respiratory illness
		9230 children given iron, folic acid and zinc		
		8683 control children		
		Ages 1–36 months		
Veenemans et al., 2011	2 × 2 Factorial trial	145 children given zinc only	Tanzania, intense malaria transmission	When data was analyzed by iron status at baseline, multi-nutrient supplementation increased the overall number of malaria episodes in children with iron deficiency by 41%, whereas multi-nutrient supplementation had no effect on the number of malaria episodes among children who were iron-replete at baseline
		148 children given both zinc and multi-nutrients (including iron)		
		146 children given multi-nutrients (including iron) without zinc		
		148 children given placebo		
		Ages 6–60 months		
Zlotkin et al., 2013	Cluster randomized, double blind	967 children given micronutrient powder with iron	Ghana, intense malaria transmission	Malaria incidence was significantly lower in the iron group compared with the no iron group during the intervention period (risk ratio [RR], 0.87; 95% CI, 0.78–0.96). In secondary analyses, these differences were no longer statistically significant after adjusting for baseline iron deficiency and anemia status overall (RR, 0.87; 95% CI, 0.75–1.01)
		991 children given micronutrient powder without iron	Insecticide treated bednets provided at enrollment	
		Ages 6–35 months	
				Subgroup analysis of 704 children who had anemia at baseline and for whom additional blood samples were obtained at the end of the intervention period found only a small mean increase in hemoglobin in the iron group (mean change of 0.08 g/dL measured), indicating that the micronutrient powder had limited efficacy in this trial
Esan et al., 2013	2-arm, double-blind, randomized	100 children received multivitamins plus iron	Malawi, intense malaria transmission	Children who received iron had a better CD4 percentage response at 3 months, but an increased incidence of malaria at 6 months (incidence rate, 120.2 vs. 71.7; adjusted incidence rate ratio [aIRR], 1.81 [95% CI, 1.04–3.16]; *p* = 0.04), especially during the first 3 months (incidence rate, 78.1 vs. 36.0; aIRR, 2.68 [95% CI, 1.08–6.63]; *p* = 0.03)
		96 children received multivitamins alone		
		HIV infected children aged 6–59 months with moderate anemia (Hgb = 7.0–9.9 g/dL); 3 months of treatment, 6 months follow up		
**CHILDREN—OBSERVATIONAL STUDIES**				
Nyakeriga et al., 2004	2 Cross sectional studies	Study 1:	Kenya, intense malaria transmission	Incidence of clinical malaria was significantly lower among children with iron deficiency anemia (incidence-rate ratio [IRR], 0.70; 95% confidence interval [CI], 0.51–0.99; *P* < 0.05)
		Iron replete (*n* = 95)		
		Iron deficient (*n* = 78)		
		Study 2:		
		Iron replete (*n* = 104)		
		Iron deficient (*n* = 91)		
		Ages 8 months-8 years		
Gwamaka et al., 2012	Longitudinal	785 children monitored for 3 years	Tanzania, intense malaria transmission	Iron deficiency anemia at routine, well-child visits significantly decreased the odds of subsequent parasitemia (23% decrease, *p* < 0.001) and subsequent severe malaria (38% decrease, *p* = 0.04). Iron deficiency anemia was also associated with 60% lower all-cause mortality (*p* = 0.04) and 66% lower malaria-associated mortality (*p* = 0.11)
Jonker et al., 2012	Longitudinal	727 children monitored for 1 year	Malawi, intense malaria transmission	Children with iron deficiency anemia at baseline had a lower incidence of malaria parasitemia and clinical malaria during a year of follow-up; adjusted hazard ratios 0.55 (95% CI:0.41–0.74) and 0.49 (95% CI:0.33–0.73), respectively
**PREGNANT WOMEN—OBSERVATIONAL**				
Kabyemela et al., 2008	Cross sectional	445 pregnant women (120 primigravidae, 112 secundigravidae, and 213 multigravidae)	Tanzania, intense malaria transmission	Iron deficiency decreased the risk of placental malaria
Senga et al., 2011	Case-Control	Pregnant women (112 cases with placental malaria, 110 controls with no evidence of placental infection)	Malawi, intense malaria transmission	Iron deficiency decreased risk of acute, chronic and past placental malaria. The association was greater in the multigravidae group

While these clinical studies and meta-analyses have been indispensable for determining the relationship between host iron status and malaria risk, it is not clear how iron deficiency protects and why iron supplementation increases risk. Immunity to malaria and high prevalence of genetic traits such as G6PD deficiency and hemoglobinopathies in the study populations limit the capacity of clinical studies to parse out causation. Furthermore, relatively little is known with regards to the role host iron plays in malaria pathogenesis. Iron impacts a broad range of biological processes that have the potential to shape malaria pathogenesis. As a result, even with the most ideal of clinical study designs; the prerequisite knowledge of which aspects of malaria pathogenesis should be studied is largely absent. A better grasp on the underlying biological principals that govern (i) the protection of iron deficiency against malaria and (ii) the increased risk of malaria associated with iron supplementation is critical for managing iron supplementation campaigns in malaria endemic regions.

## Biological importance of iron

Iron is an essential nutrient for nearly every living organism including humans and the malaria parasite. Iron impacts a broad range of biological processes; including host and parasite cellular function, erythropoiesis and immune function. The capacity of iron to fluctuate between two oxidation states, ferrous (Fe^2+^) and ferric (Fe^3+^), makes it indispensable for many critical biological processes, including DNA replication, cellular respiration, and oxygen transport. However, the same useful biphasic properties of iron which make it indispensable also contribute to its high cytotoxicity. As a result the human host tightly regulates iron availability and usage.

Access to iron is particularly important in the context of host-pathogen interactions. When confronted with infection and inflammation the human host reallocates its iron reservoirs in an effort to deprive invading pathogens of iron. The human protein hepcidin—a rheostat of systemic iron homeostasis—signals the body to decrease absorption of iron in the proximal duodenum and orchestrates the movement of iron from serum into storage within the liver and macrophages (Roy, 2013). As a result of reduced serum iron, erythropoiesis—a process exquisitely sensitive to iron levels—slows in the face of infection as well as inflammation. The human host's active reduction in bioavailable iron protects against a wide range of pathogens (Armitage et al., 2011). Not surprisingly, as many pathogens require access to host iron sources to survive and grow, pathogens have evolved sophisticated iron acquisition systems, and the iron acquisition systems of many bacterial and fungal species have been well described (Skaar, 2010). By comparison how the malaria parasite acquires, regulates, and utilizes iron remains a mystery.

## Iron metabolism in the malaria parasite

Iron is essential for the survival of the malaria parasite. The parasite multiplies 8–32 times in the course of a single intra-erythrocytic lifecycle. Iron is an essential cofactor for the DNA replication enzyme ribonucleotide reductase, and as a result iron is required to fuel this rapid intra-erythrocytic proliferation (Rubin et al., 1993). Iron is also utilized by the parasite for pyrimidine (Krungkrai et al., 1990; Van Dooren et al., 2006) and heme biosynthesis (Sato and Wilson, 2002; Dhanasekaran et al., 2004; Sato et al., 2004; Nagaraj et al., 2008, 2009, 2010, 2013). As with the human host, the malaria parasite must also balance its need for iron against the cytotoxicity of iron.

The malaria parasite metabolizes host hemoglobin in its acidic digestive vacuole in order to acquire necessary amino acids; however, as discussed below, the parasite does not utilize the iron in host heme. Plasmodium aspartic and cysteine proteases degrade host hemoglobin and release large quantities of toxic iron-laden heme (Goldberg et al., 1990; Subramanian et al., 2009). Apicoblast parasites neutralize the cytotoxic heme produced during hemoglobin metabolism by sequestering the heme in an inert crystal, hemozoin (Rudzinska et al., 1965; Chugh et al., 2013). Despite neutralizing a substantial portion of host heme into hemozoin, some residual heme remains free and becomes oxidized, generating free oxygen radicals (Francis et al., 1997). The parasite possesses powerful thioredoxin and glutathione systems to maintain intracellular redox equilibrium (Jortzik and Becker, 2012). However, even when these redox systems are functioning at full capacity, oxidative stress significantly increases as the parasite matures and replicates within host erythrocytes (Fu et al., 2010). In fact, many antimalarials, including artemisinin, appear to target the parasite's ability to detoxify reactive oxygen species (ROS) (Rosenthal and Meshnick, 1996; Klonis et al., 2013; Ariey et al., 2014). For example, it was recently found that mutations in *PF3D7_1343700* (Kelch) can confer resistance to artemisinin. The authors speculate that these mutations cause a disruption of the parasite's ability to detoxify ROS because the efficacy of artemisinin depends on its ability to generate oxygen radicals and some kelch-containing proteins in other organisms have been shown to be involved in the regulation of cytoprotection (Ariey et al., 2014).

Given the relationship between iron, heme, and ROS, it is possible that perturbations in host iron regulation might also affect the malaria parasite's redox equilibrium. Iron responsive proteins (IRPs) and their accompanying iron responsive elements are critical for maintaining cellular iron homeostasis in the human host. IRPs and iron responsive elements are responsible for mobilizing iron when demands are high and moving iron into storage when excess iron may promote ROS formation (Hentze et al., 2010). Loyevsky et al. identified and characterized a *P. falciparum* IRP, the expression of which was affected by iron starvation as well as iron supplementation (Loyevsky et al., 2001, 2003; Hodges et al., 2005). However, a search of gene databases failed to identify *Plasmodium* homologs of ferritin, ferroportin, metallothione, a ferrioxamine-based transport system or ferredoxin or siderophore biosynthesis pathways—all proteins and processes utilized by other organisms to acquire, regulate, and store iron (Scholl et al., 2005). Clearly, much remains unknown regarding parasite iron biology.

## Iron chelators and their contribution to the elucidation of malaria iron biology

Realizing the importance of iron for the malaria parasite, researchers have invested extensive time and effort into the investigation of the antimalarial activity of iron chelating agents. These studies have also provided insight into malaria parasite iron biology. In contrast to mammalian cells, which are sensitive to millimolar concentrations of iron chelators, erythrocytic stage malaria parasites are sensitive to micromolar concentrations of iron chelators *in vitro* and in animal models (Cabantchik et al., 1996). The cytotoxicity of iron chelators is dependent upon the stage of intra-erythrocytic maturation of the malaria parasite and the hydrophobicity of the iron chelator (Lytton et al., 1994). For example, the hydrophilic chelator hydroxamate-based deferoxamine (DFO) has cytostatic activity against the ring stage and cytotoxic activity against the late trophozoite and schizont erythrocytic stages of the parasite (Whitehead and Peto, 1990; Lytton et al., 1994; Cabantchik et al., 1999).

The cytotoxicity of iron chelators against the malaria parasite suggests that the mechanism of action of iron chelators is more complex than simple iron deprivation. Alternative mechanisms have been suggested for some chelators, including the direct inhibition of parasite ribonucleotide reductase activity (Lederman et al., 1984; Lytton et al., 1994). Furthermore, as iron chelators can modulate host immune function, iron chelator antimalarial activity may be a result of modification of the host immune response (Golenser et al., 2006; Li et al., 2012).

Caution must be taken when considering the use of iron chelators to inform our understanding of the biological relationship between iron deficiency and malaria infection. The evidence that iron chelators do more than merely deprive the parasite of iron introduces potential confounding factors into studies that utilize iron chelators as a model for iron deficiency. Furthermore, most iron chelators cannot chelate iron associated with heme, ferritin, or transferrin. Because the iron saturation of each of these host iron reservoirs are reduced in iron deficiency, iron chelators are not suitable for studying the effect of host iron reduction on the malaria parasite.

That said, evidence that chelation of chelatable extracellular and intra-erythrocyte iron does not impact erythrocytic stage *P. falciparum* growth, suggests that chelatable host iron is not necessary for the erythrocyte stage of infection (Scott et al., 1990). Furthermore, work by Moormann et al. shows that parasite nuclear and mitochondrial transcripts decrease in the presence of the iron chelator DFO (Moormann et al., 1999). These results are consistent with a normal cellular response to iron deprivation. In conclusion, iron chelators are obviously indispensable in the study of iron biology. However, in the case of malaria caution must be taken.

## Host iron reservoirs available to erythrocytic stage malaria

**Figure 1:**
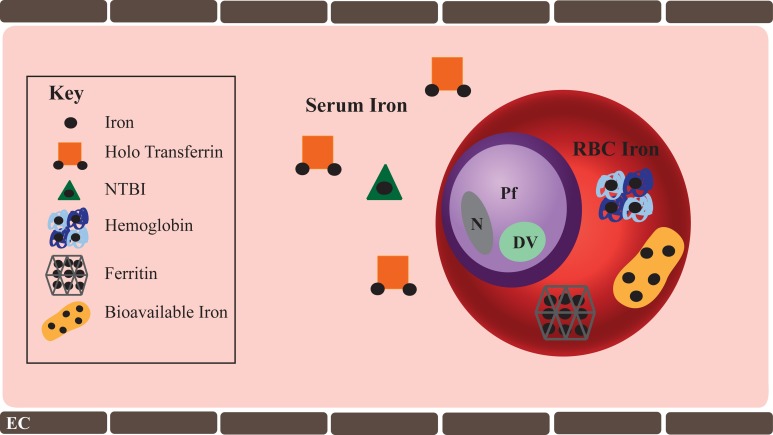
**Host Iron available to erythrocytic stage *P. falciparum***. Host iron immediately available to the erythrocytic stage of *P. falciparum* include serum and intra-erythrocytic iron. Serum iron ranges from 10 to 27 μM. Transferrin bound iron is the predominant form of iron in the serum, though trace amounts of non-transferrin bound iron (NTBI) are present. In some pathologic conditions such as hemochromatosis, NTBI may be significantly greater. While iron deficiency anemia is characterized by a significant decline in serum iron. RBC iron is found within hemoglobin (20 mM), ferritin (0.7 nM), and as bioavailable iron (1–10 μM). Iron deficiency anemia significantly reduces RBC iron, specifically hemoglobin iron. Shown in the figure are: *Pf*, *P. falciparum*; DV, digestive vacuole; N, parasite nucleus; and EC, endothelial cell.

It is inarguable that iron is essential to erythrocytic stage malaria and therefore possible that alterations in host iron levels may tip the balance between inhibiting or promoting parasite growth and virulence. Consequently, the question of how the parasite acquires host iron becomes central. A healthy iron-replete human has 3–4 total grams of iron, which is distributed in hemoglobin contained within circulating RBCs (2.5 g), in iron containing proteins (400 mg), in serum bound to transferrin (3–7 mg), and in storage proteins such as ferritin (1 g). Host iron reservoirs available to erythrocytic stage malaria parasite include: (1) transferrin and non-transferrin bound iron (NTBI) in the serum and (2) intra-erythrocytic iron contained within hemoglobin, ferritin, as well as trace amounts freely bioavailable iron in the RBC cytosol (Figure 1).

Iron deficiency affects these host iron reservoirs by significantly reducing the availability of both serum iron and intra-erythrocytic iron. Iron supplementation results in brief spikes in serum iron levels (Schümann et al., 2012, 2013), but has little immediate effect on intra-erythrocyte iron. However, approximately 2 weeks following iron supplementation, average intra-erythrocyte iron levels slowly begin improving as new iron-replete RBCs enter into circulation. It is well-documented that virulence of many bacteria is directly associated with the availability of host iron, and as a result iron supplementation can exacerbate infections (Doherty, 2007). Whether described changes in serum and intra-erythrocyte iron stores affect erythrocytic stage malaria infection remains unknown.

## The relationship between serum iron and erythrocytic stage malaria

The relationship between host serum iron and parasitized RBCs (pRBCs) is especially intriguing (Table 2). Because transferrin has an extremely high affinity for iron (10^23^M^−1^ at pH 7.4), NTBI is scarce in healthy individuals. There is strong evidence that transferrin associates with pRBCs but not uninfected RBCs. Work by Pollack et al. shows that pRBCs take up Fe^59^ bound to human transferrin, and a recent publication by our own group demonstrates that incubation of pRBCs with transferrin and ferric citrate increases the bioavailable iron in pRBCs (Pollack and Fleming, 1984; Clark et al., 2013). The idea that the parasite is able to acquire transferrin bound iron is further supported Surolia et al. who demonstrated that gelonin toxicity toward *P. falciparum* is 25 times greater when the gelonin is bound to transferrin (Surolia and Misquith, 1996). Moreover, Fry et al. report transferrin reductase activity associated with pRBCs but not uninfected RBCs (Fry, 1989). Additionally, two groups have reported the identification of a *P. falciparum* transferrin receptor in the RBC membrane of pRBCs (Haldar et al., 1986; Rodriguez and Jungery, 1986). However, a later study by Pollack et al. concluded that transferrin binding of pRBCs is non-specific (Pollack and Schnelle, 1988), and additional studies were unable to detect any acquisition of transferrin bound iron by pRBCs (Peto and Thompson, 1986; Sanchez-Lopez and Haldar, 1992).

**Table 2:** **Relationship between host serum iron and *P. falciparum***.

**Study**	**Major findings**
**STUDIES SUPPORTING TRANSFERRIN MEDIATED DELIVERY OF IRON TO pRBCs**	
Pollack and Fleming, 1984	- pRBCs take up more iron from transferrin than uninfected RBCs
Rodriguez and Jungery, 1986	- FITC labeled holo-transferrin traverses from the pRBC surface to the parasitophorous vacuole - Internalization of holo-transferrin is most active in early trophozoite stage pRBCs - A 93 kD parasite protein inserted into the RBC membrane binds human holo-transferrin
Haldar et al., 1986	- A 102kD schizont stage parasite protein inserted into the RBC membrane binds human holo-transferrin
Pollack and Schnelle, 1988	- Twice as much human holo-transferrin associates with pRBCs than uninfected RBCs - Human holo-transferrin binding to pRBCs is non-specific
Fry, 1989	- RBC membranes of pRBCs possess diferric transferrin reductase activity, uninfected RBC membranes do not - pRBC diferric transferrin reductase activity increases as the parasite matures from the ring to trophozoite stage
Surolia and Misquith, 1996	- Human transferrin conjugated to the toxin gelonin selectively binds trophozoite stage pRBCs - Toxicity of gelonin to erythrocytic stage *P. falciparum* is 25 times greater when linked to human transferrin
Clark et al., 2013	- Addition of holo-transferrin to trophozoite stage pRBCs increases the bioavailable iron content of pRBCs but not uninfected RBCs
**STUDIES REFUTING TRANSFERRIN MEDIATED DELIVERY OF IRON TO pRBCs AND STUDIES SHOWING SERUM IRON DOES NOT AFFECT *P. FALCIPARUM* GROWTH**	
Peto and Thompson, 1986	- pRBCs do not acquire iron from holo-transferrin - Depletion of iron from *P. falciparum in vitro* culture media does not reduce parasite growth - Addition of iron to *P. falciparum in vitro* culture media reduced parasite growth
Scott et al., 1990	- Restriction of iron chelator DFO to *P. falciparum in vitro* culture media does not affect parasite growth
Sanchez-Lopez and Haldar, 1992	- pRBCs do not take up iron from human holo-transferrin - Depletion of human transferrin from culture media does not affect erythrocytic stage parasite growth
**STUDIES SUPPORTING ACQUISITION OF NTBI BY pRBCs**	
Peto and Thompson, 1986	- pRBCs take up NTBI
Sanchez-Lopez and Haldar, 1992	- pRBCs take up of free, non-transferrin bound iron (NTBI), but not any more than uninfected RBCs - pRBC NTBI acquisition is time, concentration, and temperature but not energy dependent
Clark et al., 2013	- Addition of ferric citrate (NTBI) to trophozoite stage pRBCs increases the bioavailable iron content of pRBCs but not uinfected RBCs

Despite strong evidence that transferrin associates with pRBCs, neither iron depletion nor iron supplementation of malaria culture media has any observable effect on parasite growth (Peto and Thompson, 1986; Scott et al., 1990; Sanchez-Lopez and Haldar, 1992; unpublished data Clark et al.). These results challenge the idea that serum iron, specifically transferrin bound iron, contributes to the protection of iron deficiency from malaria and the increased risk of malaria associated with iron supplementation. Yet, it should be noted that malaria culture media contains tenfold less iron than human sera and all existing studies have utilized culture adapted *P. falciparum* laboratory lines. It is possible laboratory lines have adapted to an iron-starved extracellular environment. Furthermore, because hemoglobin is an essential nutrient for erythrocytic stage malaria, it is impossible to “starve” the parasite of iron *in vitro* and this may in turn limit the ability to study the effect of serum iron on *P. falciparum*.

## The relationship between intra-erythrocytic iron and erythrocytic stage malaria

Much less is known about the ability of the malaria parasite to access intra-erythrocytic iron (Table 3). An individual RBC contains 100 fg (20 mM) of iron, the majority of which is contained within hemoglobin. It is estimated that if the parasite were able to access only 1% of this hemoglobin iron all of its iron demands would be fulfilled (Hershko and Peto, 1988; Gabay and Ginsburg, 1993). However, as discussed above, the parasite incorporates the majority of heme released as a result of hemoglobin digestion into hemozoin (Chugh et al., 2013). Despite identification of a *Plasmodium* heme oxygenase-like protein, which would facilitate release of iron from host heme (Okada, 2009), the parasite does not exhibit enzymatic heme oxygenase activity nor possess a canonical heme oxygenase pathway (Sigala et al., 2012). Even without inherent heme oxygenase activity, it remains possible that non-enzymatic mechanisms release enough iron from trace heme to meet the iron requirements of the parasite. Possible mechanisms include heme breakdown by glutathione or hydrogen peroxide, the conditions for which are predicted to exist within erythrocytic stage parasites (Ginsburg et al., 1998; Loria et al., 1999). However, as the parasite synthesizes heme *de novo*, it does not seem likely that the parasite draws iron from host heme (Nagaraj et al., 2013).

**Table 3:** **Relationship between RBC iron and *P. falciparum***.

**Study**	**Major findings**
**RBC HEMOGLOBIN**	
Rudzinska et al., 1965	- *P. falciparum* metabolizes host RBC hemoglobin - *P. falciparum* inserts host heme into hemozoin
Okada, 2009	- *P. falciparum* has a heme oxygenase homolog
Sigala et al., 2012	- *P. falciparum* lacks both heme oxygenase activity and a canonical heme oxygenase pathway
Loria et al., 1999	- Hydrogen peroxide degrades host heme under conditions that are analogous to the microenvironment of the parasite food vacuole
**RBC FERRITIN—UNKNOWN**	
**RBC BIOAVAILABLE IRON—UNKNOWN**	

In addition to hemoglobin, RBCs contain residual amounts of biovaialble iron (1–10 μM) as well as iron stored within ferritin (0.7 nM), and it is possible that the parasite is capable of utilizing one or both of these erythrocyte iron reservoirs. Currently, however, there is no reported evidence to either support or refute these possibilities (Scholl et al., 2005). However, despite a lack of evidence that the parasite accesses host intra-erythrocytic iron, recent work by our group has shown that pRBC bioavailable iron content increases as the parasite matures from ring stage to schizont. This observation suggests that iron is released from some form of storage as the parasite develops within host RBCs (Clark et al., 2013). Whether the iron is released from parasite or host storage remains an open question.

Although the precise host iron source(s) the malaria parasite acquires remains unclear, all the potential host iron reservoirs (serum and intra-erythrocyte) available to erythrocytic stage malaria are affected by iron deficiency as well as iron supplementation. Therefore, it is reasonable to hypothesize that iron deprivation and excess iron contribute to the relationship between host iron and malaria risk observed in the clinical studies discussed earlier. That said, even during iron deficiency, the erythrocytic stage of the parasite inhabits the most iron rich environment in the human body. As such it is alternatively possible that neither iron deficiency nor iron supplementation perturb iron reservoirs enough to significantly impact the parasite.

## Microcytic iron deficient RBCs and malaria

In addition to affecting host iron reservoirs, iron deficiency also induces changes in RBC physiology. One such difference between iron-replete and iron-deficient RBCs is the substitution of zinc for iron in hemogloblin when iron is limiting. This results in zinc protoporphoryin IX levels ten times higher in iron deficient as compared to iron-replete RBCs (Wong et al., 1996). As zinc protoporphoryin IX inhibits hemozoin extension *in vitro*; it is reasonable to hypothesize that that elevated zinc protoporphoryin IX in iron deficient erythrocytes provides protection against malaria infection by impeding parasite growth (Iyer et al., 2003).

Additional changes to RBC physiology caused by iron deficiency include: microcytosis, greater susceptibility to oxidative stress, reduced ATP content, and decreased deformability (Yip et al., 1983; Acharya et al., 1991; Nagababu et al., 2008; Brandão et al., 2009). Furthermore, iron deficient RBCs experience enhanced eryptotic cell death (Kempe et al., 2006). The altered physiology of microcytic iron deficient RBCs may therefore protect against erythrocytic stage malaria infection. Research by Koka et al. indicates that propagation of the erythrocytic stage of *P. falciparum* strain BinH is reduced in iron deficient RBCs (Koka et al., 2007). However, earlier work by Luzzie et al. observed abnormal parasite morphology but no difference in the growth of *P. falciparum* strain UPO in iron deficient as compared to iron-replete RBCs (Luzzi et al., 1990). The differences between these studies may be explained by the use of different *P. falciparum* isolates which feasibly could have different sensitivities to iron deficient RBCs.

Accelerated host clearance of iron deficient pRBCs is an additional explanation for the protection afforded by iron deficiency against malaria. Results from two studies that examined malaria infection in iron deficient mice both observed a higher clearance rate of pRBCs in iron deficient as compared to iron-replete mice (Koka et al., 2007; Matsuzaki-Moriya et al., 2011). Specifically, Matsuzaki et al. observed elevated phagocytosis of pRBCs in iron deficient as compared to iron-replete mice, and proposed that the increased phagocytosis rate may be attributable to greater phosphatidylserine levels on iron deficient pRBCs as compared to iron-replete pRBCs. Koka et al. similarly observed greater phosphatidylserine levels on *P. falciparum* human iron deficient pRBCs. Ultimately, these limited data suggests that iron deficiency may provide protection against malaria infection by both impeding erythrocytic stage malaria growth and increasing phagocytosis of iron deficient pRBCs. However, only further investigation will reveal the true relationship between iron deficient RBCs and *P. falciparum*.

## Perturbations in erythropoiesis and malaria

**Figure 2:**
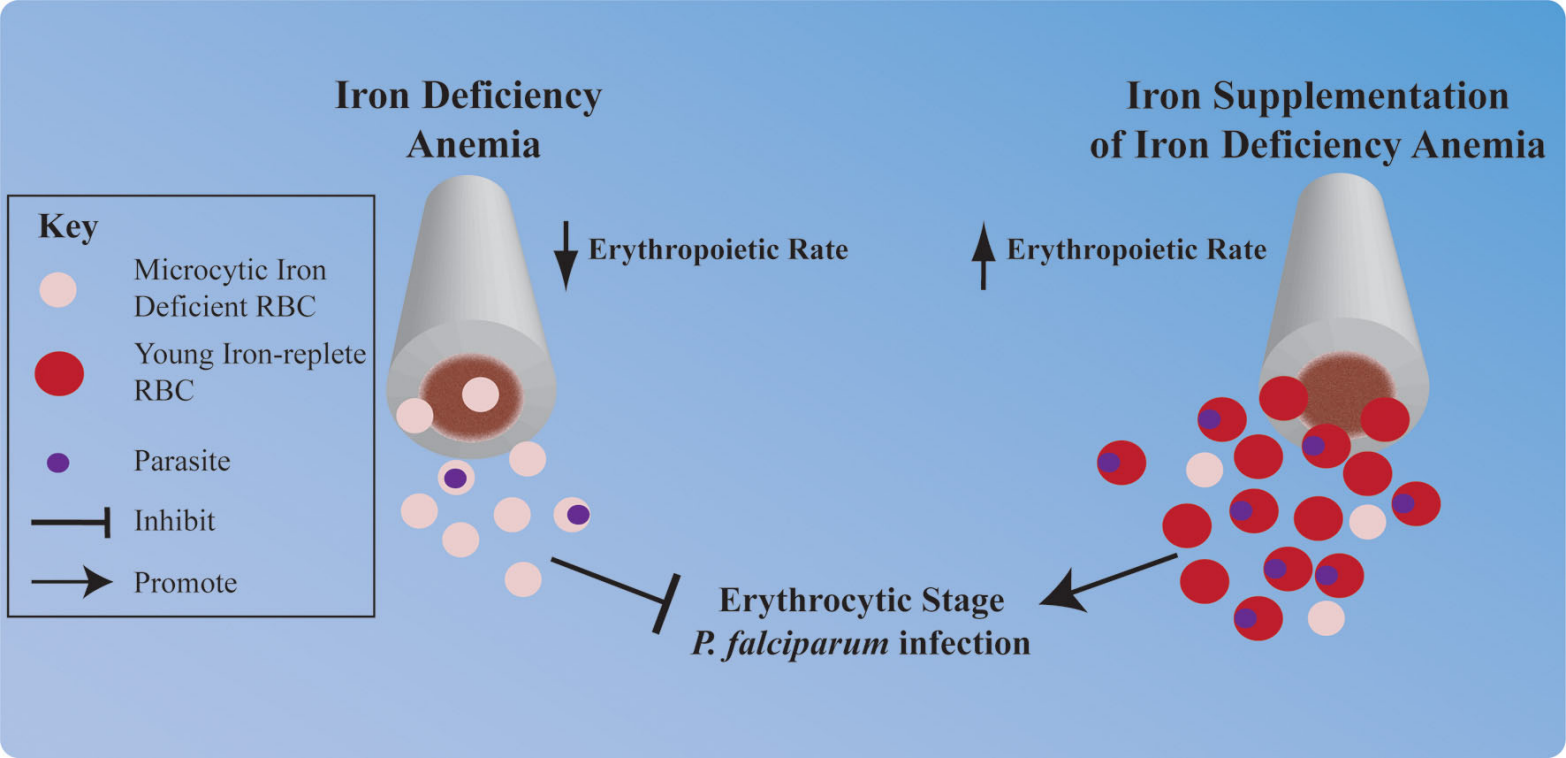
**Hypothesized impact of iron deficiency anemia and iron supplementation on *P. falciparum* erythrocytic infection**. Iron deficiency anemia and iron supplementation each profoundly influence human erythropoiesis, and this may influence erythrocytic stage malaria infection. Iron deficiency induced reduction in the erythropoietic rate and synthesis of microcytic iron deficient RBCs may provide protection against *P. falciparum* infection. Conversely, stimulation of the human host's erythropoietic rate by iron supplementation and subsequent replacement of microcytic iron deficient RBCs with young iron-replete RBCs may increase an individual's risk of erythrocytic stage *P. falciparum* infection.

In the absence of sufficient iron for heme synthesis, the human host's erythropoietic rate decreases. Conversely, iron supplementation of individuals with iron deficiency anemia results in a strong erythropoietic response; because the body attempts to recover RBC numbers and replace less viable iron deficient RBCs (Figure 2). It is well-known that *P. vivax* exclusively infects the very youngest RBCs (reticulocytes). However, *P. vivax* is not the only *Plasmodium* species that prefers young RBCs. In fact many species of *Plasmodium*, including *P. falciparum*, preferentially infect young RBCs, and furthermore young RBC support greater parasite replication than more mature RBCs (Wilson et al., 1977; Pasvol et al., 1980; Lim et al., 2013). Thus, significant elevation in the erythropoietic rate could put an individual at increased risk of erythrocytic stage *P. falciparum* infection. Tian et al. have investigated this hypothesis in the context of pregnant women, who are at greater risk of malaria infection than their non-pregnant counterparts and experience increased erythropoietic rates to meet the oxygen demands of the growing fetus. The authors report that *P. falciparum* growth is significantly greater in the on average younger RBCs taken from pregnant women as compared to the on average older RBCs taken from non-pregnant women (Tian et al., 1998).

Murine models have additionally been used to shed light on the relationship between erythropoiesis and malaria infection. Interestingly, when Chang et al. manipulated the timing of erythropoiesis during the course of a malaria infection it was observed that reticulocytosis early in infection significantly increased infection and morbidity, while reticulocytosis late in infection decreased mortality (Chang et al., 2004). These observations are consistent with recent work by Zhao et al. showing that lipocalin 2, which is elevated during malaria infection, provides protection from malaria infection in mice by limiting reticulocytosis (Zhao et al., 2012).

Furthermore, mathematical modeling by Cromer et al. makes several key predictions that support a role for erythropoiesis in driving the protection from malaria associated with iron deficiency anemia and increased risk associated with iron supplementation. First, their model predicts that low reticulocyte production rate—as would be observed in iron deficiency—in combination with a parasite that prefers reticulocytes, could result in a less severe infection. Second, high reticulocyte production—as would be observed in iron deficient individuals responding to iron supplementation—could increase severity of malaria infection (Cromer et al., 2009). These results indicate that limiting reticulocytosis early in infection is important for limiting erythrocytic stage malaria infection and further support the hypothesis that iron supplementation-induced reticulocytosis significantly increases the risk of erythrocytic stage *P. falciparum* infection.

Together, these observations provide insight into potential cellular mechanisms contributing to the protection of iron deficiency against malaria, and the increased risk of malaria associated with iron supplementation. With regard to iron deficiency, altered RBC physiology may limit *P. falciparum* propagation within iron deficient RBCs and increase clearance of iron deficient pRBCs. Furthermore, the reduced erythropoietic rate and subsequent reduction in an iron deficient individual's hematocrit may additionally contribute to protection. Conversely, the increased erythropoietic rate triggered by iron supplementation paired with the preference of *P. falciparum* for young RBCs may be partially responsible for the increased risk of malaria infection that is associated with iron supplementation.

## Conclusions and future questions

Overall, the available evidence supports a link between (i) iron deficiency and protection from malaria infection and (ii) iron supplementation and increased risk of malaria. However, there is still much to be learned. Furthermore, study of the competition between the malaria parasite and the human host for iron can serve as a translational model to identify critical molecular mechanisms of *P. falciparum* pathogenesis (see questions in Table 4). Most importantly, however, such research will help the global health community reach their goal of devising a strategy for safely administering iron supplementation in malaria endemic regions.

**Table 4:** **Questions for future translational research**.

**PARASITE**
How does the malaria parasite regulate iron?
What host iron sources are utilized by the malaria parasite?
Does the malaria parasite store iron?
Are parasite virulence factors regulated by iron?
Can merozoites sense host intra-erythrocytic iron?
**RELATIONSHIP BETWEEN PARASITE AND HOST**
Is iron limited enough during iron deficiency or in such excess following iron supplementation to respectively inhibit and exacerbate erythrocytic stage *P. falciparum* infection?
Do iron deficiency and iron supplementation affect erythrocytic stage *P. falciparum* microvascular adhesion, or host endothelial cell activation?
Do iron deficiency or iron supplementation impact parasite *var* gene expression or PfEMP1 protein levels on the RBC membrane?
Are there specific strains of *P. falciparum* that are better equipped to infect people with iron deficiency?
What is the effect of host iron deficiency and iron supplementation on *P. falciparum* gametocytogenesis?
What are the effects of changing RBC population dynamics on malaria infection?
How are the host innate and adaptive immune responses to malaria affected by iron deficiency and iron supplementation?
Is anemia of inflammation protective against malaria?
How does the presence of iron deficiency anemia modify the effects of HbS, HbC, or HbE on parasite growth, maturation, microvascular adhesion, or endothelial cell activation?
How do other malaria-protective polymorphisms, such as type O blood group antigen and glucose-6-phosphate dehydrogenase (G6PD) deficiency, interact with iron deficiency in mitigating malaria pathogenesis?

## Author contributions

Martha A. Clark, Morgan M. Goheen, and Carla Cerami wrote and edited the manuscript. All authors have read and approved the final manuscript.

### Conflict of interest statement

The authors declare that the research was conducted in the absence of any commercial or financial relationships that could be construed as a potential conflict of interest.
